# Supplementary material for: A Global Screen for Assembly State Changes of the Mitotic Proteome by SEC-SWATH-MS
Source: Cell Syst. 2020 Feb 26;10(2):133–155.e6. doi: 10.1016/j.cels.2020.01.001 (PMC7042714; doi:10.1016/j.cels.2020.01.001)

Q7Z6J0 | SH3R1_HUMAN | SH3RF1 KIAA1494 POSH RNF142 SH3MD2
Monomer MW [kDa]: 93.129 Monomer expected elution fraction: 41

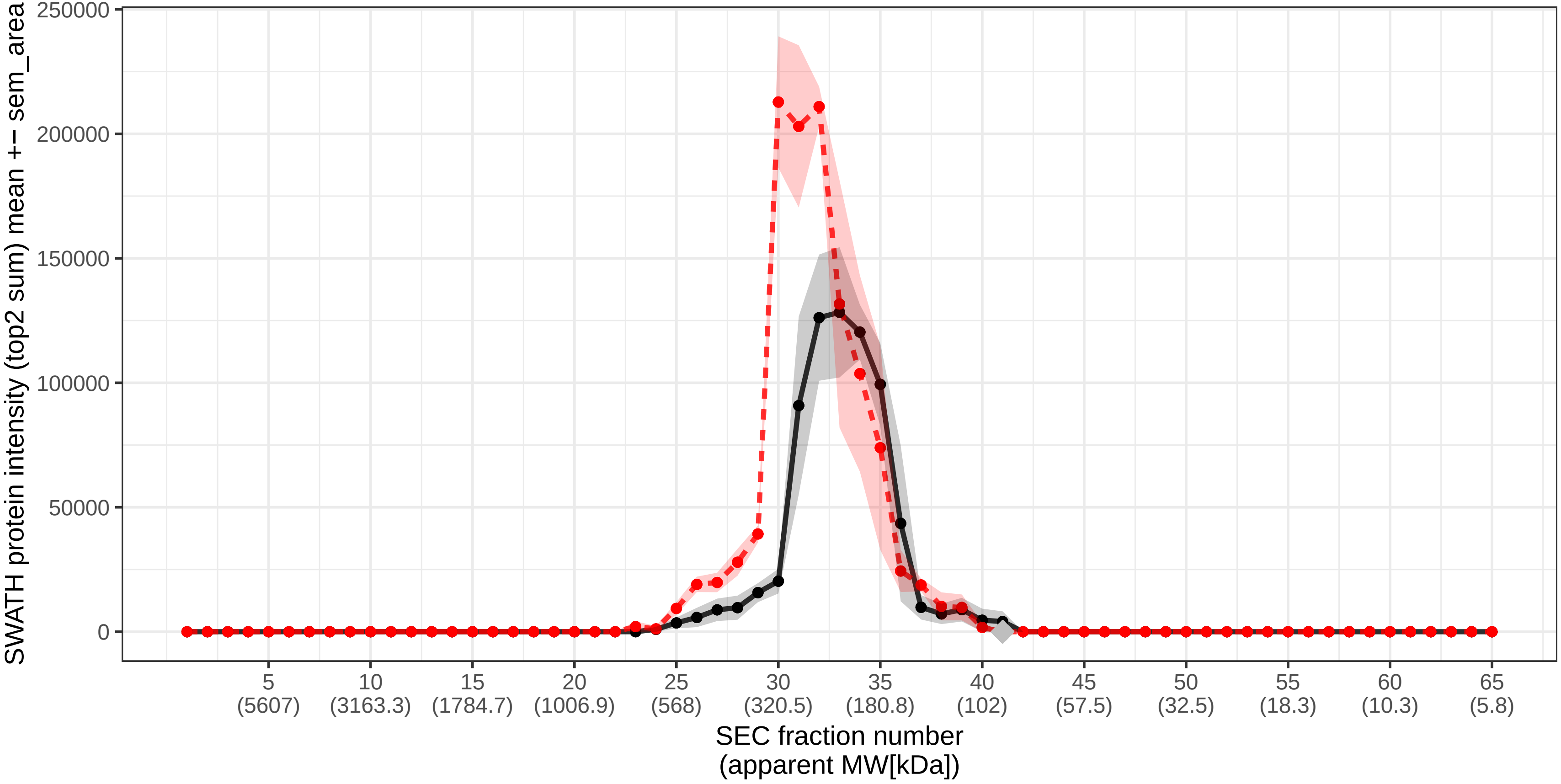

Supplement: Data S1. SEC-SWATH-MS Protein Chromatograms, Related to Figure 1 [file mmc6.zip › SECchrom_Q7Z6J0_SH3R1_HUMAN_SH3RF1_KIAA1494_POSH_RNF142_SH3.pdf]
